# A Case of Physiologic Enhancement of Scarpa′s Ganglia Mimicking Bilateral Vestibular Schwannomas in a Patient With Atypical Meningioma With NF2 Mutation

**DOI:** 10.1155/crra/1815413

**Published:** 2026-05-04

**Authors:** Elleana A. Paradise, Justin A. Schmidgall, Prachi Dubey, David S. Baskin, Bin S. Teh, Ivo W. Tremont-Lukats, Steve H. Fung

**Affiliations:** ^1^ School of Engineering Medicine, Texas A&M University, Houston, Texas, USA, tamu.edu; ^2^ Department of Radiology, Houston Methodist Hospital, Houston, Texas, USA, houstonmethodist.org; ^3^ Warren Alpert Medical School of Brown University, Providence, Rhode Island, USA, brown.edu; ^4^ Department of Neurosurgery, Houston Methodist Hospital, Houston, Texas, USA, houstonmethodist.org; ^5^ Weill Cornell Medical College, New York, New York, USA, cornell.edu; ^6^ Naresh K. Vashisht College of Medicine, Texas A&M University, Bryan, Texas, USA, tamu.edu; ^7^ Houston Methodist Research Institute, Houston, Texas, USA, houstonmethodist.org; ^8^ Kenneth R. Peak Brain and Pituitary Treatment Center, Houston Methodist Hospital, Houston, Texas, USA, houstonmethodist.org; ^9^ Department of Radiation Oncology, Houston Methodist Hospital, Houston, Texas, USA, houstonmethodist.org; ^10^ Department of Neuro-Oncology, AdventHealth Orlando, Orlando, Florida, USA, adventhealth.com

**Keywords:** meningioma, NF2 mutation, Scarpa′s ganglion, vestibular ganglion, vestibular schwannoma

## Abstract

We followed a 36‐year‐old man with an atypical meningioma with somatic NF2 mutation and invasion into the bone, temporalis muscle, and pterygopalatine fossa, treated with surgical resection and adjuvant radiation therapy. His medical history included medulloblastoma treated with resection and adjuvant radiation therapy and a WHO Grade 1 meningioma treated with gross total resection. A brain MRI during routine follow‐up revealed small enhancing foci in both internal auditory canals (IACs), which prompted concern for bilateral vestibular schwannomas and suspect NF2‐related schwannomatosis (NF2‐SWN). However, the lesions were symmetric, fundal, and nonnodular on high‐resolution constructive interference in steady‐state (CISS) imaging, with smooth vestibular nerve contours, long‐term interval stability, and a lack of clinical symptoms, favoring physiologic enhancement of the vestibular ganglia (Scarpa′s ganglia) rather than vestibular schwannomas. Recognition of these distinguishing features also helped avoid associating the IAC findings with the known NF2 mutation, which was determined to be somatic. While germline NF2 mutations (NF2‐SWN) often lead to meningiomas and vestibular schwannomas, somatic NF2 mutations are common in sporadic meningiomas and should not suggest NF2‐SWN. This case highlights the need to correlate small enhancing IAC foci with morphology on CISS MRI, symmetry, longitudinal stability, and NF2 pathology to avoid misdiagnosis of enhancing Scarpa′s ganglia as vestibular schwannomas, as misdiagnosis can lead to increased patient anxiety and unnecessary follow‐ups.

## 1. Introduction

The vestibular ganglion (or Scarpa′s ganglion) is located near the posterior internal auditory canal (IAC) fundus and is the site of bipolar neuronal cell bodies for both superior and inferior vestibular nerves [1, 2]. While its physiologic enhancement is typically imperceptible on imaging, variable enhancement may mimic vestibular schwannomas, creating a diagnostic challenge [2]. It is important to distinguish small enhancing foci in the IACs from vestibular schwannomas and other etiologies like vascular enhancement or partial volume averaging to determine appropriate treatment and management [3]. Vestibular schwannomas are benign tumors that commonly arise from similar regions to Scarpa′s ganglia, further complicating differentiation, and can lead to nerve damage and hearing loss, often requiring surgical intervention [4]. In general, physiologic enhancement of Scarpa′s ganglia is favored when the findings are symmetric, centered at the fundus, and lack discrete nodularity or mass effect on high‐resolution constructive interference in steady‐state (CISS) imaging. In contrast, vestibular schwannomas are more likely to appear as focal nodular lesions with corresponding contour abnormality of the vestibular nerve.

This case was also confounded by a meningioma with a known NF2 mutation. NF2‐related schwannomatosis (NF2‐SWN) is a rare, inherited disorder that may lead to benign tumor growths, especially vestibular schwannomas. In the absence of NF2‐SWN, bilateral vestibular schwannomas are extremely rare [2]. In this patient, the NF2 pathology determined that the mutation was somatic, not germline, and confined to the meningioma. Somatic NF2 mutations are noninherited genetic disorders caused by mutations in the NF2 gene on Chromosome 22 and commonly arise in meningiomas [5–10].

## 2. Case Presentation

This case report follows a 36‐year‐old man with a remote history of medulloblastoma, a more recent history of meningioma with recurrence, primary hyperparathyroidism, and small enhancing lesions in bilateral IACs. His oncological history began at the age of 22 when he was diagnosed with cerebellar medulloblastoma that was subsequently resected and irradiated. Surveillance imaging over the next 10 years showed no recurrence.

A follow‐up MRI revealed a left perisylvian extra‐axial mass measuring over 1 cm. He underwent left frontotemporal craniotomy with gross total resection of the mass. Pathology showed meningioma, WHO Grade 1, with some areas having increased Ki‐67 proliferation index. A month later, he presented with hypercalcemia and elevated parathyroid hormone. The patient was diagnosed with primary hyperparathyroidism secondary to right superior parathyroid adenoma, and this was treated by parathyroidectomy.

Approximately 18 months after the initial craniotomy, the patient reported a palpable bump in his left temporal region anterior to the left ear. Follow‐up CT imaging showed an ill‐defined enhancing soft tissue mass overlying the left frontotemporal craniotomy that was inseparable from the left temporalis muscle, concerning for recurrent extracranial meningioma with a maximum dimension of 3.3 cm. Preoperative MRI confirmed an extensive tumor involving the craniotomy bone flap, left temporalis muscle, and left pterygopalatine fossa. Repeat left frontotemporal craniotomy with excision of surrounding tissue was performed with pathology confirming atypical meningioma, associated with *NF2* and *SMARCA4* mutations. Immunohistochemical stains showed a Ki‐67 proliferation index up to 15%. Following surgery, the patient underwent adjuvant radiation therapy.

**Figure 1:**
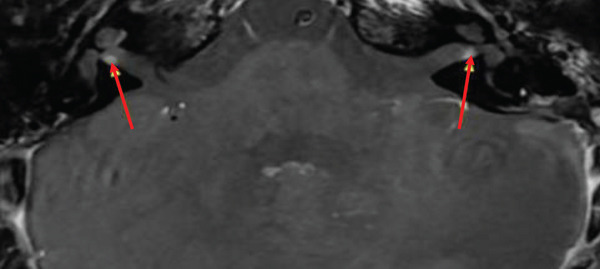
Contrast‐enhanced T1‐weighted MRI shows symmetric foci of enhancement in the fundi of bilateral IACs (two red arrows). Physiologic enhancement of Scarpa′s ganglia is favored when the findings are symmetric and centered at the fundus, versus the asymmetrical location, size, and morphology typically seen in vestibular schwannomas.

**Figure 2:**
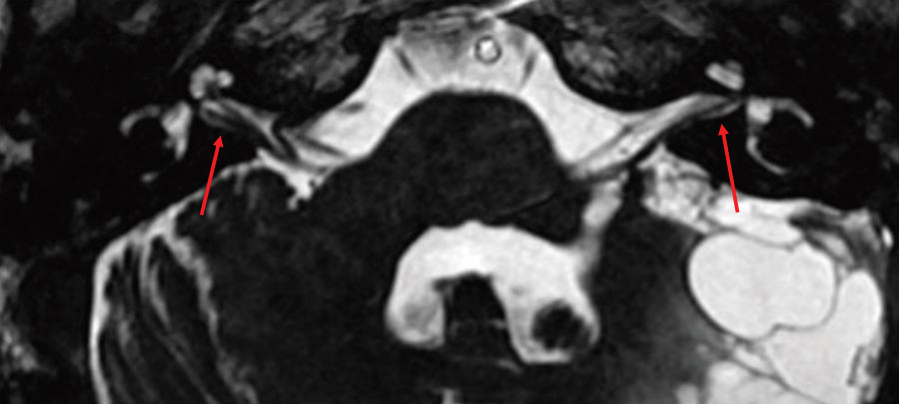
High‐resolution constructive interference in steady‐state (CISS) imaging shows normal smooth contours of fundal vestibular nerves (two red arrows) without corresponding nodularity. Physiologic enhancement of Scarpa′s ganglia is favored when vestibular nerve contours are smooth without nodularity, versus the rounded contours and discrete nodularity seen in vestibular schwannomas.

A follow‐up brain MRI that included high‐resolution postcontrast T1‐weighted imaging revealed symmetrical small foci of enhancement near the fundus of bilateral IACs, measuring approximately 2 mm (Figure 1). At the time, this was concerning for small bilateral vestibular schwannomas and, by extension, NF2‐SWN given the recent diagnosis of atypical meningioma with NF2 mutation. However, high‐resolution CISS MRI showed smooth vestibular nerve contours without nodularity (Figure 2), suggesting a different etiology, such as physiologic enhancement of Scarpa′s ganglia. Retrospective review of prior brain MRIs showed variable visualization of the small foci of enhancement in bilateral IACs, in part due to partial volume averaging from thicker image slices used. Nevertheless, the 2‐mm fundal enhancing foci were present and stable over 10 years of follow‐up imaging. Table 1 shows the brain MRI without and with contrast, IAC protocol, used at our institution.

**Table 1:** Brain MRI without and with contrast, IAC protocol.

Sequence	TR	TE	FA	NEX	FOV	Matrix	In‐plane resolution	Slice thickness
3D CISS	8 ms	2 ms	55°	1	180 × 180 mm	512 × 512	0.35 × 0.35 mm	1 mm
T1WI TSE precontrast	813 ms	15 ms	111°	2	180 × 180 mm	512 × 512	0.35 × 0.35 mm	2 mm
T1WI TSE postcontrast fat sat	885–1030 ms	16 ms	111°	2	180 × 180 mm	512 × 512	0.35 × 0.35 mm	2 mm

*Note:* CISS acquired in the axial plane and reformatted in coronal and sagittal planes. T1WI TSE precontrast acquired in the axial plane. T1WI TSE postcontrast with fat saturation acquired in axial and coronal planes. Additional sequences include focused DWI using a non‐EPI technique covering the IAC.

## 3. Discussion

When the bilateral foci were observed in the fundus of the IACs, there was immediate concern for vestibular schwannomas. However, the patient did not exhibit any clinical symptoms typically associated with vestibular schwannomas, such as hearing loss, vertigo, or tinnitus [4]. Furthermore, serial imaging over a 10‐year period demonstrated stability of the lesions, which led us to consider possible mimics like vascular enhancement, partial volume averaging, or physiologic enhancement of Scarpa′s ganglia.

Physiologic enhancement of Scarpa′s ganglia is poorly understood and highly variable [1]. It has been hypothesized that the variable enhancement corresponding to Scarpa′s ganglion may be the result of differing amounts of capillaries within the arachnoid villi or pillars present within the IAC cistern [3, 11]. Small, rounded areas of enhancement near the fundus of the IACs are not uncommon but require careful correlation with high‐resolution imaging to distinguish between normal anatomical variants and true pathological processes such as schwannomas [3]. While the “gold standard” for detecting pathology in the IACs is high‐resolution contrast‐enhanced T1‐weighted MRI, a combination of axial CISS and coronal T2‐weighted MRI has been shown as an effective, safer, and less costly alternative with excellent diagnostic accuracy [2, 12]. Normal Scarpa′s ganglia on CISS MRI typically appear as smooth, subtle fusiform dilatations, whereas vestibular schwannomas exhibit more discrete, rounded contours [2]. In this patient, high‐resolution CISS MRI revealed smooth contours of the fundal vestibular nerves, which suggested physiologic enhancement from Scarpa′s ganglia rather than true vestibular schwannomas. By recognizing these distinguishing features, we avoided unnecessary treatment or intervention of a benign, nonpathological phenomenon.

This case was further complicated by the patient′s history of an atypical meningioma with an NF2 mutation. NF2 mutations can be either inherited germline or somatic. NF2‐SWN (formerly neurofibromatosis Type 2 [13]) is an inherited disorder caused by mutations in the NF2 gene on Chromosome 22, leading to the development of multiple tumors primarily due to the loss of function of the tumor suppressor protein Merlin encoded by the NF2 gene [5, 6, 14]. These tumors include schwannomas (especially bilateral vestibular schwannomas), meningiomas, and ependymomas. Approximately 50%–75% of patients with NF2‐SWN develop one or more meningiomas [5–7]. Biallelic NF2 inactivation is a key driver of tumorigenesis and is observed in a majority of sporadic meningiomas and nearly all schwannomas [9, 15, 16].

On the other hand, somatic NF2 mutations are noninherited genetic disorders and occur in sporadic tumors, like meningiomas. Approximately 30%–60% of meningiomas harbor somatic NF2 mutations, especially in fibrous and transitional subtypes, as well as in higher grade tumors and radiation‐induced meningiomas [5–10]. Misinterpreting a somatic NF2 mutation as a germline mutation and therefore making a false positive diagnosis of NF2‐SWN could reinforce misinterpreting the small enhancing lesions in bilateral IACs as vestibular schwannomas, especially when preliminary imaging findings are suspect, as they were in this patient. If the imaging findings were incorrectly interpreted as bilateral vestibular schwannomas, it would have been conceivable to believe that the patient had NF2‐SWN that led to the development of his meningioma and bilateral vestibular schwannomas. However, by confirming the NF2 pathology as somatic, we avoided attributing the NF2 status to the IAC findings since bilateral vestibular schwannomas are extremely rare in the absence of NF2‐SWN [2]. It is likely that his somatic NF2 mutation was confined to the meningioma tumor cell population and was possibly radiation‐induced from his history of radiation therapy.

## 4. Conclusion

This case demonstrates some of the diagnostic challenges associated with interpreting small enhancing lesions in bilateral IACs and the importance of integrating clinical, imaging, and genetic data for accurate diagnosis. While the presence of the enhancing lesions initially suggested bilateral vestibular schwannomas, the absence of clinical symptoms, the symmetry and fundal location of the lesions, their long‐term stability, and the smooth nerve contours without nodularity on CISS MRI collectively pointed toward physiologic enhancement of Scarpa′s ganglia rather than vestibular schwannomas. It is important for radiologists to be aware of these distinguishing features, especially in the context of a confounding NF2 mutation. One should also be aware that somatic NF2 mutation is the most common genetic alteration in meningiomas and should not be confused for germline NF2 mutation that would suggest NF2‐SWN‐induced schwannomas. Misinterpreting enhancing lesions in the IACs for vestibular schwannomas can lead to misdiagnosis of NF2‐SWN, resulting in increased patient stress and anxiety and unnecessary follow‐ups. For cases of small enhancing lesions in IACs, there is a need to correlate findings with nodularity on CISS imaging to make the diagnosis of either vestibular schwannoma or another benign etiology like physiologic enhancement of Scarpa′s ganglia.

## Funding

No funding was received for this manuscript.

## Conflicts of Interest

The authors declare no conflicts of interest.

## Data Availability

The data that support the findings of this study are available on request from the corresponding author. The data are not publicly available due to privacy or ethical restrictions.
